# Metal Chelation Following Inappropriate Testing: Approach to Non-Medically Indicated Therapy Requests: Ethics in Practice

**DOI:** 10.1007/s13181-026-01149-1

**Published:** 2026-08-03

**Authors:** Neeraj Chhabra, Sean McCann

**Affiliations:** Department of Emergency Medicine, Section of Medical Toxicology, University of Illinois Chicago, 808 S Wood Street, 5th Floor, Chicago, IL 60612 USA

**Keywords:** Chelation, Non-medically indicated therapy, Clinical ethics, Physician patient relationship

## Abstract

Requests for chelation therapy following inappropriate or scientifically unsound testing in medical toxicology practice raise important ethical tensions between patient autonomy, professional integrity, and the therapeutic relationship. The central tension is how medical toxicologists should respond when patients strongly request non–medically indicated therapies that lack evidence of benefit and carry potential risk. We argue that while respect for patient autonomy requires earnest engagement with patient concerns, it does not obligate physicians to provide therapies they believe are scientifically unsupported or potentially harmful. Ethical care in these situations requires balancing patient autonomy with nonmaleficence and responsibilities to preserve professional integrity and public trust. Patients pursuing alternative therapies are sometimes motivated by prior experiences of dismissal, unresolved symptoms, and distrust of conventional medicine. Reflexive refusal or adversarial communication may further erode trust and increase the likelihood of patients pursuing unsafe therapies elsewhere. We propose that medical toxicologists should approach these encounters with transparent communication, acknowledgment of medical uncertainty and patient suffering, and continued therapeutic engagement even when declining requested treatment. Maintaining a therapeutic alliance while avoiding non-indicated interventions is challenging in practice but promotes long-term patient health and preserves trust, both between physician and patient and between the public and profession of medical toxicology.

## Case

A 43-year-old female presents to an outpatient medical toxicology practice following months of fatigue and “brain fog”, described as a sense of decreased mental acuity and trouble concentrating. She has been on leave from her employment for the past month due to her symptoms. She was recently diagnosed with heavy metal poisoning after a chelator challenge test performed at an alternative medicine clinic. She was previously treated for heavy metal poisoning at the same clinic five years prior and experienced symptom improvement following the prescribed therapy. The previous therapy was a proprietary formulation of multiple products, including an unknown chelation agent, and she incurred significant out-of-pocket expenses for the treatment. She is requesting chelation therapy for treatment of heavy metal poisoning and sought a medical toxicology practice so therapy would be covered by her insurance.

Question: How should a medical toxicologist respect patient autonomy when a patient requests a non–medically indicated therapy, particularly when the intervention carries risks but is strongly desired?

## Ethical Analysis

This case highlights tensions between patient’s autonomy and a physician’s duty to provide evidence-based, nonharmful care. It also reflects an increasingly common challenge in contemporary medical practice: patient requests for unproven alternative therapies that sometimes arise from a distrust of conventional medicine [1]. For medical toxicologists, the challenge is not only determining whether a requested therapy is medically appropriate, but also how to best advocate for scientifically justified care without further eroding trust.

A comprehensive review of post-chelation challenge testing and chelation therapy is beyond the scope of this discussion. However, challenge testing has not been found to reliably diagnose disease, may lead to potentially harmful therapies, and its practice has been discouraged by professional organizations including the American College of Medical Toxicology [2–4]. Requests for non-medically indicated therapies are often driven by multiple overlapping factors. Patients may feel dismissed by prior clinicians, frustrated by unexplained symptoms, skeptical of traditional healthcare institutions and the pharmaceutical industry, or have had previous positive experiences with self-treatment or alternative medicine [5]. These factors are amplified by readily accessible medical information online and via social media, some of which has questionable accuracy [1, 6]. Importantly, patients seeking alternative therapies are not only searching for symptom relief but often validation and acknowledgement of real suffering. They may approach conventional medical encounters anticipating conflict or dismissal. Recognizing these motivations and the potential for heightened tensions is important to provide compassionate care and reduce adversarial interactions.

A request for a non-medically indicated treatment highlights tensions between core principles of medical ethics: autonomy, beneficence, and nonmaleficence [7]. While patient autonomy represents the core ethical principle in modern medicine, there are important limitations. Autonomy is generally understood as a negative right in that treatment options can be refused by a patient but not compelled [8]. Patient autonomy does not obligate physicians to provide interventions that lack indication and may be harmful [9]. Even when decisions are informed, physicians are not obligated to provide treatments they believe to be professionally inappropriate. A common example is a patient request for opioid analgesia without indication. Acquiescence ignores duties to avoid undue risk and patients may be better served by a thorough investigation for etiologies of symptoms.

As with any proposed treatment, a careful weighing of the benefits and potential risks should be performed within the context of uncertainties and the patient’s goals and values. Although non-medically indicated therapies carry no medical benefits, there may still be relational benefits to the encounter itself, especially when a patient feels heard, respected, and valued. While these relational benefits do not justify exposing patients to unnecessary risk, acknowledgement helps frame care as more than a simple refusal for inappropriate treatment.

Providing non-medically indicated chelation therapy also undermines public trust in medical toxicology by legitimizing unsupported practices. A physician’s obligations extend beyond the individual encounter to the integrity of the profession and the care of future patients [10]. By maintaining the public perception of medical toxicologists as thoughtful stewards of potentially harmful therapies, medical toxicologists can advance population health even when disappointing individual patients.

Medical toxicologists must balance these concerns while building and maintaining a therapeutic alliance with the patient. The physician should avoid reflexive dismissal and alienation but instead explore patient concerns in a nonjudgmental manner [1]. A thorough assessment for true toxicologic or medical disease within their scope of practice should be accompanied by soliciting expectations from the patient. When diagnostic limitations or uncertainties are known, as they often are in medical toxicology, they should be acknowledged honestly [6, 11]. Maintaining a therapeutic alliance, even if denying therapy, requires continued engagement and the offer of appropriate alternatives. The physician should emphasize commitment to ongoing symptom management and further evaluation, when appropriate, rather than ending the relationship after disagreement. Preserving trust increases the likelihood of future follow-up and adherence to evidence-based recommendations. This continued relationship may reduce the risk of harm if the patient later considers unsafe or exploitative treatments.

## Conclusion

Ethically appropriate care includes relational responsibility and not merely a refusal to provide non-medically indicated therapy. Requests for chelation after inappropriate diagnostic testing require medical toxicologists to balance patient autonomy with nonmaleficence, professional integrity, and trust. The medical toxicologist should neither acquiesce to potentially harmful treatments nor respond dismissively. Instead, they should provide clear evidence-based guidance while preserving a compassionate therapeutic alliance focused on the patient’s long-term wellbeing.

## Data Availability

Data sharing not applicable to this article as no datasets were generated or analyzed.

## Declarations

### Disclaimer

This article is designed to provide an accessible, clinically relevant ethics discussion tailored to the practicing medical toxicologist. The case is intended to illustrate a dilemma in clinical practice and is fictionalized. The discussion focuses on ethical considerations and does not constitute legal guidance.
